# A cluster of cases of severe fever with thrombocytopenia syndrome bunyavirus infection in China, 1996: A retrospective serological study

**DOI:** 10.1371/journal.pntd.0006603

**Published:** 2018-06-25

**Authors:** Jianli Hu, Chao Shi, Zhifeng Li, Xiling Guo, Yanhua Qian, Wenwen Tan, Xian Li, Xian Qi, Xiaoju Su, Minghao Zhou, Hua Wang, Yongjun Jiao, Changjun Bao

**Affiliations:** 1 Jiangsu Provincial Center for Disease Control and Prevention, Nanjing, China; 2 Wuxi Municipal Center for Disease Control and Prevention, Wuxi, China; 3 Yixing County Center for Disease Control and Prevention, Yixing, China; 4 Zhangzhu Township Health Center, Yixing, China; 5 Jiangsu Provincial Commission of Health and Family Planning, Nanjing, China; Beijing Institute of Microbiology and Epidemiology, CHINA

## Abstract

**Background:**

A cluster of eleven patients, including eight family members and three healthcare workers with fever and thrombocytopenia occurred in Yixing County, Jiangsu Province, China, from October to November 1996. However, the initial investigation failed to identify its etiology. Severe fever with thrombocytopenia syndrome (SFTS) is an emerging infectious disease caused by SFTS bunyavirus (SFTSV), which was first discovered in 2009. The discovery of novel SFTSV resulted in our consideration to test SFTSV on the remaining samples of this cluster in September 2010.

**Methodology/Principal findings:**

We retrospectively analyzed the epidemiological and clinical data of this cluster. The first case, one 55-year-old man with fulminant hemorrhagic diseases, died on October 14, 1996. His younger brother (the second case) developed similar hemorrhagic diseases after nursing him and then died on November 3. From November 4 to November 15, nine other patients, including six family members and three medical staffs, developed fever and thrombocytopenia after exposure to the second case. The sera of six patients were collected on November 24, 1996. IgM antibodies against SFTSV were detected in all of the six patients’ sera using enzyme-linked immunosorbent assay (ELISA), while IgG antibodies were detected in one patient’s serum using an indirect immunofluorescence assay (IFA). We also found that IgG antibodies against SFTSV were still detected in four surviving patients’ sera 14 years after illness onset.

**Conclusions and significance:**

The mysterious pathogen of the cluster in 1996 was proved to be SFTSV on the basis of its epidemiological data, clinical data and serological results. It suggests that SFTSV has been circulating in China for more than 10 years before being identified in 2009, and SFTSV IgG antibodies can persist for up to 14 years.

## Author summary

SFTSV was first discovered in 2009. It can be transmitted through tick bites, direct contact with SFTS patients’ blood or bloody secretion, and probable aerosol transmission. SFTS was listed as one of the nine infectious diseases on the WHO priority list in 2017 because of its trend of wider distribution and rising threat imposed on global health. It is worth mentioning that a cluster of eleven patients including eight family members and three healthcare workers developed fever and thrombocytopenia in China, from October to November 1996, but the initial investigation failed to identify its etiology. A retrospective analysis was conducted in September 2010. Based on the epidemiological, clinical, and serological findings, we speculated that SFTSV was the mysterious pathogen of the cluster. Meanwhile, high-titer SFTSV IgG antibodies were detected in four surviving patients’ sera. These SFTS patients preceded two cases in Japan in 2005 reported by Kurihara *et al*., which were once regarded as “the earliest SFTS cases worldwide”. These results suggest that SFTS should have existed without being diagnosed for a long time, and SFTSV IgG antibodies can persist long for 14 years, and perhaps even a lifetime after infection.

## Introduction

Severe fever with thrombocytopenia syndrome (SFTS), an emerging haemorrhagic fever, was firstly confirmed among the rural areas in the central and eastern regions of China in 2009[1]. The main clinical features include fever, thrombocytopenia, leukocytopia, lymphadenopathy, and gastrointestinal symptoms. It has an average case-fatality rate of 12% but can be as high as 30%[2]. The causative agent, SFTS bunyavirus (SFTSV), is classified into the *Phlebovirus* genus, *Phenuiviridae* family, *Bunyavirales* order. It was once called as fever, thrombocytopenia and leucopenia syndrome virus (FTLSV)[3], or Huaiyangshan virus (HYSV)[4]. SFTSV is believed to be transmitted through tick bites[1, 5, 6], direct contact with SFTS patients’ blood or bloody secretion[7, 8], and probable aerosol transmission[9]. SFTS cases outside China were first reported in North Korea in 2009[10], South Korea in 2012[11] and Japan in 2013[12]. A closely related virus called Heartland virus was isolated from patients with similar symptoms in the United States [13]. Hence, SFTS was listed as one of the nine infectious diseases on the WHO priority list in 2017 because of its trend of wider distribution and rising threat imposed on global health.

Serological investigation showed that SFTSV infection was widespread in domestic animals (e.g. goats, sheep, cattle, dogs, etc.) and wild animals (e.g. rodent and shrews)[14, 15]. The seroprevalence of SFTSV in healthy people in China varies from 0.23% to 9.17%, depending on the investigated population and geography as well as the test reagent and methods, but only a small proportion of exposed persons develop clinical symptoms[16]. From the published documents, two SFTS cases in Japan in 2005 reported by Kurihara *et al*. have been regarded as the earliest cases in the world [17]. A recent phylogenetic study on SFTSV in China, South Korea, and Japan demonstrated that SFTSV could be divided into the Chinese clade and the Japanese clade, which may have evolved separately over time, except for the rare occasion of overseas transmission[18].These results suggest that SFTS may have existed without being identified for some time. In this study, we completed a retrospective analysis of a cluster of eleven patients with unexplained fever and thrombocytopenia in China in 1996 to determine whether SFTSV was responsible for this cluster.

## Methods

### Data and samples collection

The cluster of eleven patients with unexplained fever and thrombocytopenia occurred from October to November in 1996 in a township in Yixing County, which is located in southern Jiangsu Province of China and is characterized by hilly terrain. When the cluster was detected, public health workers were dispatched immediately to record the clinical and epidemiological information of patients and explore its causative agent. Sera of six patients were collected on November 24, 1996. Although the delay between the illness onset and sampling ranged from 9 to 20 days, all efforts were made to explore the causative agent at that time. Common pathogens including *Hantavirus*, *Crimea-Congo Hemorrhagic fever virus*, *Orientia tsutsugamushi*, *Spotted fever group rickettsiae*, *Coxiella burnetii*, *Rickettsia Prowazeki*, *Rickettsia Mooseri*, *Salmonella typhi* and other bacteria were excluded by blood culture and antibody tests in December 1996. Then, a small amount of the remaining samples were stored at a temperature of -80°C in the laboratory of Jiangsu Provincial Center for Disease Control and Prevention (JSCDC).

The discovery of novel SFTSV and impact of clinical manifestations of SFTS resulted in our consideration to test SFTSV on the remaining samples of this cluster in September 2010. Meanwhile, a retrospective investigation was conducted through interviewing the patients’ family members, neighbors and medical staffs, cross-checking several written timelines of the cluster, and collecting surviving patients’ sera.

### Laboratory testing

Acute-phase sera of SFTS patients were detected for SFTSV-specific IgM antibodies using an ELISA kit (Xinlianxin, Wuxi, China) according to the manufacturer’s protocol [19]. In the initial screening, an undiluted serum sample was used to determine whether the sample was positive for antibodies against SFTSV. Positive serum samples were further diluted in 2-fold increments starting at 1:2 for titration of antibody titers with the same assay.

SFTSV-specific IgG antibodies were detected in all human sera by IFA as previously described [20].Twenty microliters of diluted (1:2 to 1:1280) serum samples were added to the cell-spotted coverslips with viral antigens and incubated for 45 minutes at 37°C. After washing, 20μL of FITC-conjugated goat anti-human IgG (Abcam, UK) diluted 1:80 with Phosphate Buffered Saline (PBS) containing Evans Blue (1:20,000) was added for further incubation for 30 minutes at 37°C. After washing for three times, the slides were mounted in glycerin and examined under an immunofluorescence microscope.

### Ethics approval

The study was approved by the Ethics Committee of JSCDC and informed consent was obtained from the participants. All data were analyzed anonymously.

## Results

### Index case (patient A)

On October 2 1996, a 55-year-old man developed dizziness, fatigue and sore throat, followed by fever and chills on October 4. Then, he developed nausea, vomiting, hematemesis and melena on October 12. Laboratory testing revealed that he had thrombocytopenia, leukopenia, elevated serum alanine and aspartate transaminase levels, proteinuria, and hematuria the next day.

On the morning of October 14, he was admitted to the local township healthcare center. Physical examination showed conjunctival congestion, scleral icterus, ecchymosis on the back of his right hand and right wrist joints, and sporadic hemorrhagic spots on his soft palate. He was administered with dexamethasone and oxygen. He died on that evening.

Retrospective investigation of the patient’s family members revealed that he was a mine safety supervisor, and his hobby was hunting. He caught three hares in the woods near his residence approximately 30 days before illness onset. He had no wife or child. During his illness, his younger brother attended to him day and night.

### Patient B

The second case was patient A’s younger brother. Patient B not only attended to patient A during his illness, but also cleaned his body and dressed him in funeral clothes before cremation. He had sudden onset of fever, chills, and headache on October 25, 11 days after patient A’s death. He was admitted to the same township healthcare center on October 28. On November 1, he was transferred to People's Hospital of Yixing County because of the severe condition. Physical examination on admission revealed supraclavicular lymph node enlargement and hepatomegaly. On November 2, he bled from his mouth and nose, and developed neurological symptoms such as seizures and extensive skin ecchymosis. He died the next morning despite the intensive care including transfusion and hemostatic therapy.

### Patient C~ patient K

**Fig 1:**
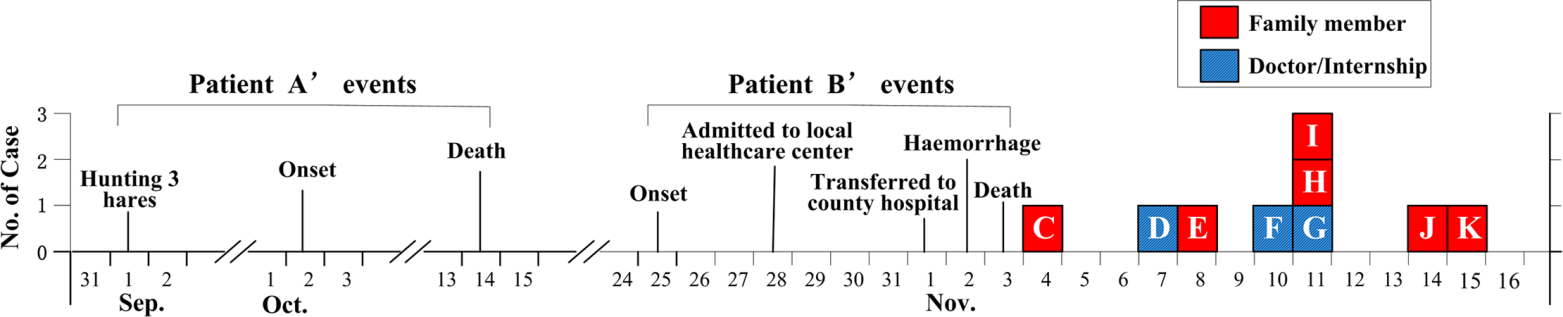
Epidemic curve shows a timeline of key events in the cluster. Capital letters designate the corresponding cases.

From November 4 to November 15, nine cases, including six family members and three medical staffs, all developed fever and thrombocytopenia. Patient C (patient B’s brother) developed symptoms on November 4 firstly, followed by patient D (patient B’s doctor), patient E (patient B’s daughter) and patient F (patient B’s doctor) on November 7, November 8 and November 10; patient G (patient B’s doctor), patient H (patient B’s elder son) and patient I (patient B’s younger son) presented with fever on November 11; The last two patients, patient J (patient B’s brother-in-law) and patient K (patient B’s nephew) developed symptoms on November 14 and 15, respectively. The mean age of these nine cases was 38.5 years (ranged from 22 to 63 years). Eight patients were male and one patient was female. Compared to the two fatal cases (patient A and B), eight follow-up cases had shorter duration from illness onset to admission with milder symptoms, and finally recovered after supportive treatment. The timeline of key events is shown in Fig 1, and all patients’ demographic and clinical information is shown in Table 1.

**Table 1:** Demographic information, clinical characteristics and serological testing results of patients involved in the cluster in Yixing County, China, 1996.

Patient	Patient A	Patient B	Patient C	Patient D	Patient E	Patient F^#^	Patient G	Patient H	Patient I	Patient J	Patient K
**Demographic information**											
Relationship	Index patient	Second patient (Index patient’s brother)	Second patient’s brother	Second patient’s Doctor	Second patient’s daughter	Second patient’s Doctor	Second patient’s Doctor	Second patient’s son	Second patient’s son	Second patient’s brother-in-law	Second patient’s nephew
Gender	M	M	M	M	F	M	M	M	M	M	M
Age	55	53	63	55	32	22	45	29	26	45	30
Occupation	Farmer	Farmer	Farmer	Doctor	Farmer	Intern doctor	Doctor	Farmer	Farmer	Farmer	Farmer
Date of onset	Oct. 2	Oct. 25	Nov. 4	Nov. 7	Nov. 8	Nov. 10	Nov. 11	Nov. 11	Nov. 11	Nov. 14	Nov. 15
**Clinical characteristics** *											
Fever (°C)	35.8 (39.6)	38.0 (40.1)	37.0 (38.5)	38.5 (39.8)	37.3 (40.0)	38.3	37.8 (39.8)	39.8 (40.1)	39.0 (39.5)	36.5 (39.0)	39.1 (40.0)
other symptoms	Haematemesis, melena and mucocutaneous hemorrhage	Haematemesis, epistaxis, skin ecchymosis and seizures	Oral mucosa petechia and hepatosplenomegaly	Myalgia,nausea,vomiting and diarrhea	Fatigue, poor appetite and hepatosplenomegaly	NA	Chill, fatigue, myalgia, and vomiting	Headache,conjunctival congestion and hepatosplenomegaly	Headache, diarrhea, congestion of throat and hepatosplenomegaly	Myalgia, backache, fatigue	Epistaxis, congestion of throat hepatosplenomegaly
White-Cell count	3.6	3.6 (2.0)	4.3 (2.8)	3.2 (3.2)	5.3 (4.5)	NA	2.0 (2.0)	4.4 (3.7)	5.6 (4.0)	2.2 (2.2)	4.2 (4.0)
Platelet count	59	73 (18)	120 (100)	30 (30)	130 (70)	NA	12 (12)	110 (110)	120 (100)	66 (30)	100 (100)
Days of hospitalization	1	7	15	6	4	NA	16	15	15	12	17
Outcome	Died/Oct. 14	Died/Nov. 3	Survival	Survival	Survival	NA	Survival	Survival	Survival	Survival	Survival
**Serological testing**											
Result of IgM-ELISA, 1996	NA	NA	1:2	1:32	NA	NA	1:2	1:8	1:4	NA	1:2
Result of IgG-IFA, 1996	NA	NA	< 1:10	1:10	NA	NA	< 1:10	< 1:10	< 1:10	NA	< 1:10
Result of IgG-IFA, 2010	NA	NA	1:80	1:640	Lost to follow-up	Lost to follow-up	1:80	Lost to follow-up	Lost to follow-up	1:80	Lost to follow-up

*Data were measured at admission (numbers in parenthesis showed peak or nadir measurement during hospitalization).

# Clinical characteristics of patient F were his complaints at the illness onset.

NA = not available.

ELISA = Enzyme-linked immunosorbent assay.

IFA = Indirect fluorescent assay.

The specimens in 1996 were collected on November 24, 1996.

The specimens in 2010 were collected on September 17, 2010.

Two doctors (patient D&G) worked in People's Hospital of Yixing County and lived in the center of Yixing County. Patient F was an intern doctor in 1996. There was no clinical information about patient F in existing records, because he returned to his college in another city for medical treatment after his illness onset. Prior to the onset of the disease, the three medical staffs had provided medical services for patient B, while six family members had participated in attending to him in People's Hospital of Yixing County. Meanwhile, these nine cases had no contact with patient A before illness onset. Retrospective interviews showed that the three medical staffs had contact with blood or bloody secretion of patient B while rescuing the critically ill patient B on the evening of November 2; patient C and patient J cleaned up his body’s blood after patient B died; Detailed exposure histories of other patients were not remembered clearly by them and their family members.

### Laboratory testing

Sera from patient C, patient D, patient G, patient H, patient I and patient K were collected on November 24, 1996. No serum was available from patient A and patient B. The time span from illness onset to sampling ranged from 9 to 20 days. SFTSV-specific IgM antibodies were detected in all of the six patients’ sera by ELISA, and SFTSV-specific IgG antibodies were detected in the sera of patient D by IFA. However, no SFTSV was isolated by using Vero, Vero-E6 and BHK 21 cell culture and no viral RNAs were detected by real-time reverse transcription PCR from these serum samples.

Sera of the four surviving patients were collected on September 17, 2010, nearly 14 years after illness onset. SFTSV IgG antibody titers were 1:80 in patient C, patient G and patient J, and 1:640 in patient D.

## Discussion

The cluster of eleven patients with unexplained fever and thrombocytopenia in 1996 occurred 14 years before the discovery of SFTSV. These cases were initially diagnosed as a viral haemorrhagic fever caused by Hantavirus or Crimea-Congo Hemorrhagic fever virus, which were known as the most common viruses causing severe hemorrhagic diseases in China, due to the main clinical manifestations including fever, thrombocytopenia and hemorrhages. However, the antibody test and nucleic acid test for these two viruses were negative and further analysis was carried out. Then, differential diagnosis including *Orientia tsutsugamushi*, *Spotted fever group rickettsiae*, *Coxiella burnetii*, *Rickettsia Prowazeki*, *Rickettsia Mooseri*, *Salmonella typhi* and other bacteria were considered, but the test results were all negative. Therefore, we suspected an outbreak of a severe transmissible infection of unknown etiology in People's Hospital of Yixing County and requested notification of all similar cases from the local medical institutions. However, there was no evidence of more cases at that time. Remaining serum samples from six cases were kept stored in a freezer at a temperature of -80°C from November 1996. To test SFTSV on these samples was considered owing to the discovery of novel SFTSV in 2009 and the impact of clinical manifestations of SFTS.

There are five reasons to extrapolate that SFTSV was the mysterious pathogen of the cluster. Firstly, all ten patients with medical records developed typical symptoms of SFTS such as fever and thrombocytopenia. Moreover, six of them had leucopenia, and two of them died with severe hemorrhage compatible with severe SFTS. Secondly, the cluster occurred in Yixing County, which was characterized by hilly terrain. Serological results showed that SFTSV had been circulating widely in Yixing County. The overall SFTSV seroprevalence in urban and rural residents in Yixing County in 2011 was 0.20% [21]. Average SFTSV seroprevalence in animal species in Yixing County in 2012 were: goats(66.8%), cattle(28.2%), dogs(7.4%), pigs(4.7%), chickens(1.2%), geese(1.7%), rodents(4.4%) and hedgehogs(2.7%)[14]. Thirdly, SFTSV infection was reported to occur from April to October annually in Jiangsu Province [22]. The index case had illness onset in October, which was in accordance with the seasonal pattern of SFTSV. Although the index case had no clear history of tick bite, he had high exposure risk for tick due to his occupation of a mine safety supervisor and his hobby of hunting. Fourthly, transmission was closely associated with blood or bloody secretion exposure from the index case or patient B, both of whom died of a fulminant febrile illness with hemorrhage. This is consistent with SFTSV transmission patterns reported in the previous literatures[7, 8, 23]. Last but not least, IgM antibodies against SFTSV were detected in the acute-phase serum samples of six patients by ELISA. Although SFTSV isolation and viral RNA detection are the gold standards for diagnosis, the appearance of anti-SFTSV IgM by ELISA is useful and has become one of the diagnostic criteria for a laboratory-confirmed SFTS case in China since the specificity and sensitivity of ELISA test is similar to those of the microneutralization assay and anti-SFTSV IgM exhibit no cross-reactivity with these antibodies to other closely related viruses such as hantavirus, Rift Valley fever virus, dengue virus, and so on[24–26]. Based on all of these findings, the cluster of eleven patients with unexplained fever and thrombocytopenia in China in 1996 was most likely caused by SFTSV.

Although one recent research by Qing-Bin Lu *et al*. found that SFTSV specific IgM antibody could be detected at a median of 9 days and remained persistent until 6 months after disease onset[27], it seems to be a theoretical concern more than a practical one (i.e., the chance of a person acquiring SFTSV infection during a given transmission season, maintaining a significant level of virus-specific IgM activity over the ensuing 6 months, and then again being re-exposed to SFTSV during the subsequent transmission season is highly unlikely, because our previous studies indicate that the seroprevalence rate of SFTSV in high risk population is less than 2% in Yixing County and the incidence of SFTSV infection is less than 5 cases/100,000 population in the highest incidence county[14, 28]). Therefore, the appearance of anti-SFTSV IgM is still a possible indicative sign of the clinical disease.

One highlight of our study was that SFTSV IgG antibody titers were detected in surviving patients with high titers 14 years after illness onset, suggesting that SFTSV IgG antibody could last for more than 10 years, perhaps even a lifetime after infection. At present, only one research about the persistence of SFTSV IgG antibodies found that SFTSV IgG antibody could be detected 3 years after infection[27]. It should be noted that the IFA used to detect IgG antibody against SFTSV in our study has good specificity and sensitivity compared to RT-PCR, and no any cross with other arbor-virus including hantavirus, and Japanese encephalitis virus, etc. The other highlight was that the cluster comprising eleven SFTS patients occurred in Yixing County, China, in 1996, which preceded the cases in Japan in 2005 reported by Kurihara *et al*. that might be mistaken as the earliest SFTS cases worldwide[17]. Our result suggests that SFTS have existed for a long time without being recognized.

Three limitations exist in our study. Firstly, no tissue or serum sample of patient B and patient F, especially of the index patient, was available for retrospective laboratory detection. Secondly, no viral RNAs were detected by real-time reverse transcription PCR from the six patients’ sera. This might be because the time of sera collection was later than the patients’ viremia period, or because the long preservation time and the sera freeze thawing resulting in viral RNA degradation. Thirdly, the cluster occurred such a long time ago that detailed disease-related exposure history could not be clearly remembered and completely recorded. Memory bias may exist in our research.

Our findings suggest that SFTSV has been circulating in China for more than 10 years before being identified and SFTSV IgG antibodies can persist for as long as 14 years.

## Supporting information

S1 Checklist STROBE checklist. (DOC)
